# Improved Performance for the Electrochemical Sensing of Acyclovir by Using the rGO–TiO_2_–Au Nanocomposite-Modified Electrode

**DOI:** 10.3389/fchem.2022.892919

**Published:** 2022-05-11

**Authors:** Xin-Yang Lu, Jing Li, Fen-Ying Kong, Mei-Jie Wei, Pei Zhang, Ying Li, Hai-Lin Fang, Wei Wang

**Affiliations:** School of Chemistry and Chemical Engineering, Yancheng Institute of Technology, Yancheng, China

**Keywords:** acyclovir, electrochemical sensor, chemically modified electrode, metallic oxide, reduced graphene oxide

## Abstract

An electrochemical sensor for sensitive sensing of acyclovir (ACV) was designed by using the reduced graphene oxide–TiO_2_–Au nanocomposite-modified glassy carbon electrode (rGO–TiO_2_–Au/GCE). Transmission electron microscopy, X-ray diffractometer, and X-ray photoelectron spectroscopy were used to confirm morphology, structure, and composition properties of the rGO–TiO_2_–Au nanocomposites. Cyclic voltammetry and linear sweep voltammetry were used to demonstrate the analytical performance of the rGO–TiO_2_–Au/GCE for ACV. As a result, rGO–TiO_2_–Au/GCE exerted the best response for the oxidation of ACV under the pH of 6.0 PB solution, accumulation time of 80 s at open-circuit, and modifier amount of 7 µl. The oxidation peak currents of ACV increased linearly with its concentration in the range of 1–100 µM, and the detection limit was calculated to be 0.3 µM (S/N = 3). The determination of ACV concentrations in tablet samples also demonstrated satisfactory results.

## Introduction

Acyclovir (2-amino-9-[(2-hydroxyethoxy) methyl]-6,9-dihydro-3H-purin-6-one, ACV), an effective antiviral drug of synthetic deoxyguanosine analog, plays an important role in the therapy of viral diseases (Clercq, 2004). However, if ACV was used in an inappropriate and excessive manner, many other adverse reactions related to kidneys and certain neurotoxicity effects will occur (Lu et al., 2012). Therefore, measuring the concentration of ACV in pharmaceuticals and biological fluids appears very significant, and a number of analytical methods have already been developed for the analysis of ACV (Yu and Xiang, 2008; Long and Chen, 2012; Ajima and Onah, 2015; Liu et al., 2015; Mulabagal et al., 2020). Electrochemical sensors based on chemically modified electrodes are ideal candidates to more traditional methods, owing to their advantages of simplicity, sensitivity, rapidity, portability, and cheapness (Tarlekar et al., 2018; Bao et al., 2021; Kong et al., 2021). In addition, electrochemical methods can provide the drug-based electrode reaction mechanism, while the redox properties of drugs offer insight into their metabolism, *in vivo* redox processes, or pharmacological activity (Özkan et al., 2003; Özkan et al., 2004).

Reduced graphene oxide (rGO) is a promising candidate for the preparation of high-performance modified electrodes because of its unique 2D structures, interesting electrocatalytic activity, and excellent conductivity (Coros et al., 2020). Moreover, these characteristics can be tailored by making rGO as the building blocks with metal oxide and metal nanoparticles (Chakraborty and Roychaudhuri, 2021). Metal oxide nanoparticles, such as TiO_2_, have garnered extensive attention in the design of a potential sensing interface due to their non-toxicity, high surface area, exceptional chemical stability, and notable electrochemical properties (George et al., 2018). Meanwhile, noble metal nanoparticles such as Au and Ag possess outstanding conductivity, remarkable electrocatalytic properties, and good biocompatibility, which make them fit as modifiers for sensor fabrication (Xiao et al., 2020). All these alluring features endow the composite of rGO–TiO_2_–Au with an improved sensing performance based on the excellent synergetic effect among them.

With regard to the aforementioned survey, we prepared the rGO–TiO_2_–Au nanocomposite-modified glassy carbon electrode (GCE) for the sensitive sensing of ACV. On the rGO–TiO_2_–Au/GCE, a significant improvement of the oxidation peak current of ACV was observed. It was endowed with the sensitive determination of ACV. On the basis of optimizing various experimental parameters, such as the pH of the supporting electrolyte, accumulation time and potential, and the modifier amount, the rGO-TiO_2_-Au/GCE showed a wide linear range and low detection limit. The selectivity, repeatability, and stability of rGO–TiO_2_–Au/GCE for the determination of ACV were also evaluated, and the results were acceptable. Finally, the rGO–TiO_2_–Au/GCE was implemented for the estimation of ACV in tablet samples, which offered excellent recovery.

## Experimental

### Chemicals and Reagents

Graphite powder, chloroauric acid (HAuCl_4_·4H_2_O), and the ACV standard powder were purchased from Aladdin Reagent Co. Ltd. Sodium citrate, NaH_2_PO_4_, Na_2_HPO_4_, H_3_PO_4_, NaOH, and other chemical reagents were purchased from Sinopharm Chemical Reagent Co., Ltd. ACV tablets (200 mg) were purchased from the local pharmacies. All other chemicals and reagents used in this work were of analytical grade and used directly. ACV stock solution (1.0 mM) was prepared by dissolving appropriate amounts of the ACV powder in ultrapure water. Before use, it was stored at 4°C in the dark to avoid any decomposition. The required concentration of ACV was made by diluting the stock solution. The supporting electrolyte was 0.1 M phosphate buffer (PB) solution. The pH varying from 5.5 to 8.0 was obtained by mixing NaH_2_PO_4_ and Na_2_HPO_4_, using H_3_PO_4_ and NaOH as reagents for pH adjusting. Ultrapure water (18 MΩ cm) obtained from a Milli-Q water purifying system was used for preparation of all the solutions.

### Apparatus and Characterization

The structure and surface morphology of the prepared products were observed by transmission electron microscopy (TEM) (JEOL JEM-2100F) operated at 200 kV. The surface composition and chemical state of the rGO–TiO_2_-–Au nanocomposites were examined by X-ray photoelectron spectroscopy (XPS) with a monochromatic Al Kα excitation source. The crystal phase of the materials was investigated by using a Bruker D8 Advance X-ray diffractometer at 40 kV with Cu Kα radiation (λ = 1.54 Å). The electrochemical performance of the nanocomposites was measured using a CHI 660E electrochemical workstation. A standard three-electrode system was engaged, which comprised a GC (bare or modified) working electrode with a diameter of 3 mm, a platinum wire counter electrode, and a 3-M KCl-saturated Ag/AgCl reference electrode. All the electrochemical experiments were carried out at room temperature with dissolved oxygen removed by a N_2_ stream.

### Synthesis of the rGO–TiO_2_–Au Nanocomposites

GO, TiO_2_, and Au nanoparticles were first prepared. GO was prepared by the oxidation of the graphite powder using a modified Hummer’s method. Experimental details were given in the literature (Kong et al., 2015). TiO_2_ nanoparticles were synthesized by the hydrothermal method using tetrabutyl titanate as the titanium source (Ya et al., 2015). Au nanoparticles were synthesized by the citrate reduction method. Details can be found in our previous work (Kong et al., 2019).

For the synthesis of rGO–TiO_2_–Au nanocomposites, 10 mg of GO was dispersed in 20 ml ultrapure water and sonicated for 30 min to obtain a uniform dispersion. Then, 5.0 ml of GO dispersion was mixed with 100 µl TiO_2_ and sonicated for 1.0 h. After adding 100 µl Au nanoparticles and ultrasonic treatment for another 0.5 h, the mixture was poured into a transparent vial and illuminated using a UV-LED spot lamp. After irradiation for 3 h, the product was collected by centrifugation and washed several times with ultrapure water.

### Fabrication of the rGO–TiO_2_–Au/GCE

The preparation process of rGO–TiO_2_–Au/GCE was as follows: initially, the GCE was carefully polished with 0.3 and 0.05 μm alumina powder in sequence on a polishing cloth to obtain a mirror-like surface. After ultrasonic cleaning in 1:1 nitric acid, ethanol, and ultrapure water for 5 min, it was dried under N_2_ flow. Then, 7 μl of rGO–TiO_2_–Au dispersion was drop-cast on the surface of GCE using a microsyringe, and it was dried naturally under a closed vessel.

### Electrochemical Measurements

The electrochemical measurements were performed in 10 ml of 0.1 M PB solution containing a certain concentration of ACV using cyclic voltammetry (CV) and linear sweep voltammetry (LSV). The accumulation step was performed at open-circuit for 80 s under stirring. After 5 s quiescence, LSV was recorded between +0.6 and +1.4 V. The oxidation peak currents were measured at 1.10 V for the quantification of ACV.

### Analysis of the Sample

A total of ten tablets of ACV were finely powdered using the agate and a mortar, and a portion of this powder was accurately weighed and dissolved in ultrapure water with ultrasonic agitation for 10 min to ensure complete dissolution. Finally, it was filtered and diluted to volume with ultrapure water. A desired volume of the sample solution was transferred to the electrochemical cell and analyzed under optimal conditions using the LSV technique. The ACV content in the tablet was calculated using the standard addition method.

## Results and Discussion

### Characterization of the rGO–TiO_2_–Au Nanocomposites

**FIGURE 1:**
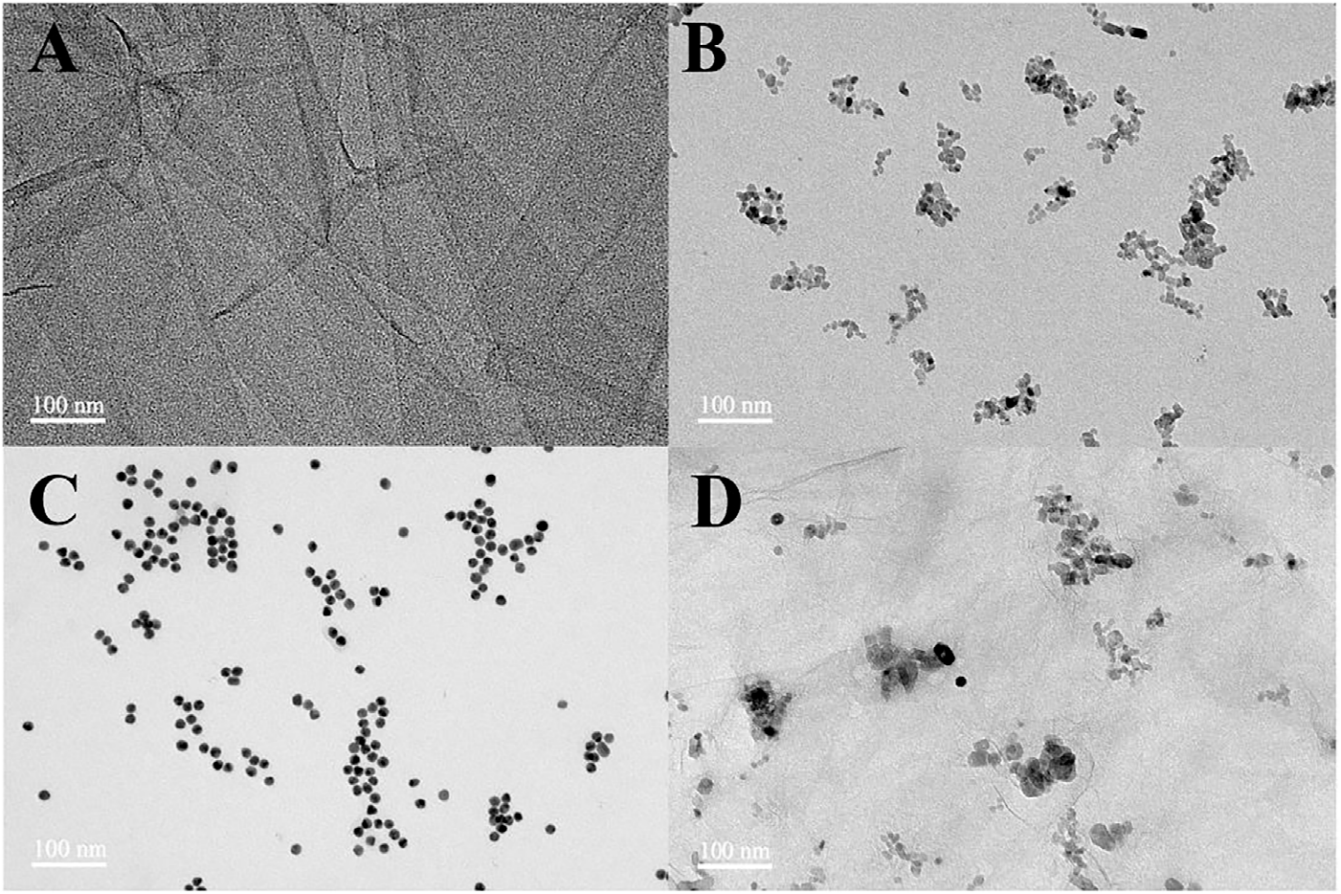
TEM images of GO **(A)**, TiO_2_ nanoparticles **(B)**, Au nanoparticles **(C)**, and rGO–TiO_2_–Au nanocomposites **(D)**.

The microstructure and morphology of the prepared nanomaterials are observed by TEM, and the obtained images are displayed in Figure 1. Figure 1A depicts the TEM micrograph of GO, in which an ultra-thin, wrinkled, and sheet-like structure is observed. The TEM image of TiO_2_ exhibits a small amount of aggregation, which is composed of spheroid nanoparticles with a diameter of about 10 nm (Figure 1B). Figure 1C shows the well-monodispersed and spherical shape of Au nanoparticles with a mean size of about 15 nm. The obtained nanocomposites maintained the 2D sheet structure as shown in Figure 1D. TiO_2_ and Au nanoparticles were loaded on the rGO sheet and accumulated along the wrinkles and edges.

**FIGURE 2:**
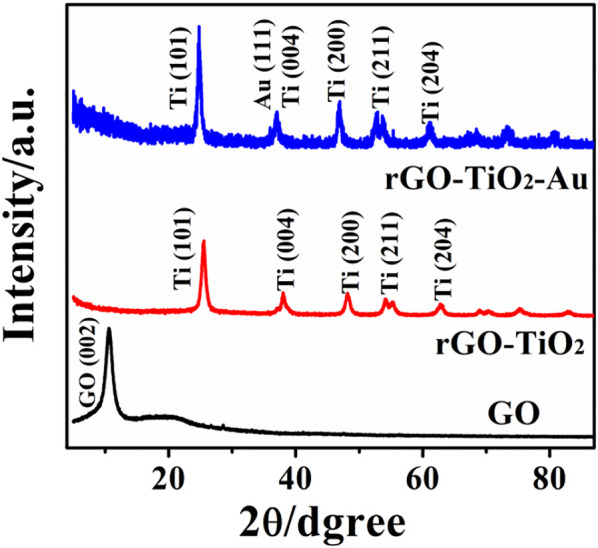
XRD patterns of GO, rGO–TiO_2_, and rGO–TiO_2_–Au nanocomposites.

XRD analysis was recorded to study crystalline characteristics of the nanocomposites, and the results are presented in Figure 2. As can be observed, GO reveals a sharp and intensive diffraction peak at 2*θ* of 11.0°, which corresponds to the (002) plane of the graphene sheets (Wang et al., 2008), indicating the formation of GO by the Hummers’ method. The XRD pattern of rGO–TiO_2_ shows the diffraction peaks at 25.3°, 37.8°, 48.0°, 55.1°, and 62.7°, which can be indexed to (101), (004), (200), (211), and (204) crystal planes of anatase TiO_2_ (JCPDS card no.21-1272) (Li et al., 2007), respectively. Meanwhile, the characteristic diffraction peak of GO disappears, indicating the successful reduction of GO via UV irradiation. According to a previous report (Kong et al., 2016; Yang et al., 2016), when TiO_2_ is exposed to UV light, the photo-induced electron–hole pairs are produced. The separated holes react with water to generate oxygen and H^+^, whereas the electrons are effectively captured by the GO substrate to reduce functional groups. The XRD pattern of the rGO–TiO_2_–Au nanocomposites is similar to rGO–TiO_2_, suggesting that the introduction of Au nanoparticles did not alter their lattices.

**FIGURE 3:**
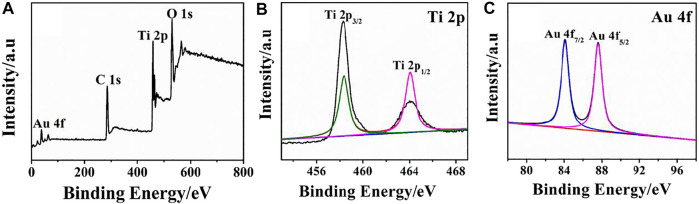
XPS survey spectra **(A)** and high-resolution spectra of Ti 2p **(B)** and Au 4f **(C)** of the rGO–TiO_2_–Au nanocomposites.

The formation of rGO–TiO_2_–Au nanocomposites and their surface features were examined by XPS, and the corresponding results are illustrated in Figure 3. The presence of major elements such as Au, C, Ti, and O from the survey spectrum conveys the successful preparation of rGO–TiO_2_–Au nanocomposites (Figure 3A). For the Ti 2p spectrum, two main peaks appeared at 459.4 and 464.1 eV fit binding energy of Ti 2p_3/2_ and Ti 2p_1/2_ (Figure 3B), confirming Ti ions occur in the form of Ti^4+^ states. The result is consistent with the reported literature (Zhu et al., 2020). In Figure 3C, Au 4f displays two peaks located at a binding energy of 84.17 and 87.82 eV, which are assigned to Au 4f_7/2_ and Ag 4f_5/2_ of metallic Au, respectively (Shukla et al., 2018).

### The Electrochemical Oxidation of Acyclovir at the rGO–TiO_2_–Au/GCE

**FIGURE 4:**
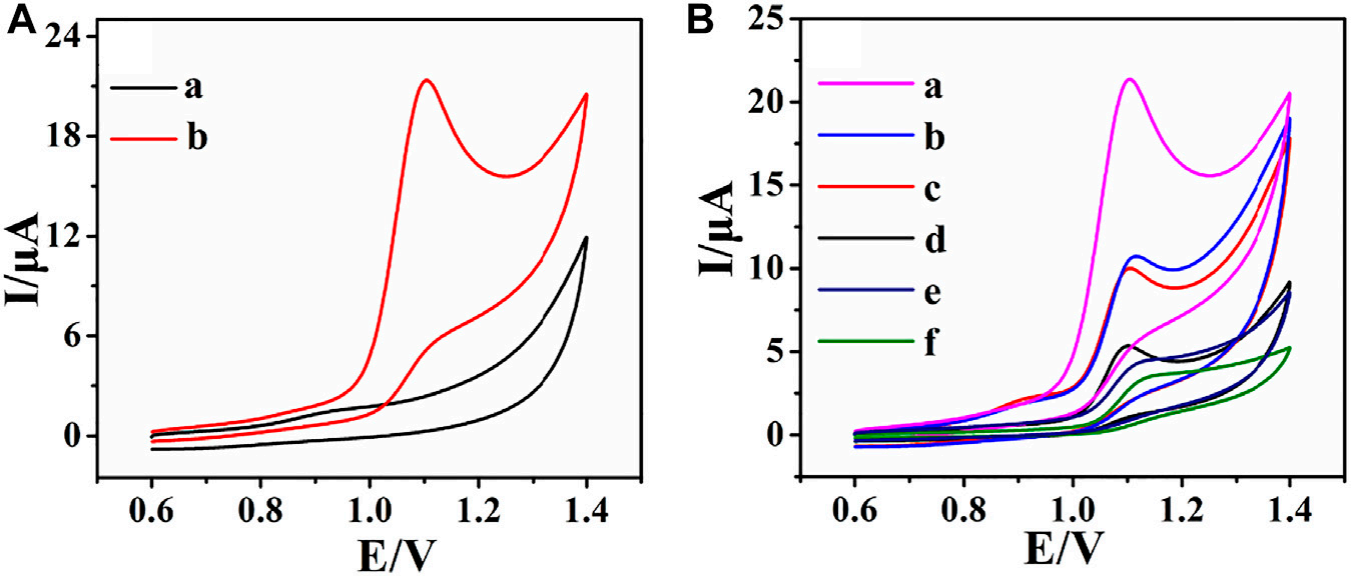
CVs of rGO–TiO_2_–Au/GCE in the absence (a) and presence (b) of 0.2 mM ACV in 0.1 M PB solution (pH 6.0) **(A)**; CVs of rGO–TiO_2_–Au/GCE (a), rGO–Au/GCE (b), rGO–TiO_2_/GCE (c), rGO/GCE (d), TiO_2_/GCE (e), and bare GCE (f) in 0.1 M PB solution (pH 6.0) containing 0.2 mM ACV **(B)**. Scan rate: 100 mV s^−1^.

The electrochemical oxidation of ACV at the rGO–TiO_2_–Au/GCE was investigated by the CV method (Figure 4). In the potential range from +0.6 to +1.4 V, the CV in the absence of ACV shows no observable redox peaks (Figure 4A, curve a). However, in the presence of ACV, a well-resolved oxidation peak is observed at about 1.10 V (Figure 4A, curve b), indicating that the oxidation peak is attributed to ACV. Furthermore, no reduction peaks are found in the reverse scan, suggesting that the electrochemical reaction is a totally irreversible process. Based on the aforementioned results and previously published literatures (Wang et al., 2013; Dorraji and Jalali, 2016; Ilager et al., 2021), a possible oxidation mechanism of ACV involves the two-electron and two-proton transfer process for the formation of 8-oxoacyclovir, which is structurally analogous to the preliminary oxidation product of guanine (Supplementary Figure S1).

The electrochemical behavior of ACV at various electrodes included rGO–TiO_2_–Au/GCE (a), rGO–Au/GCE (b), rGO–TiO_2_/GCE (c), rGO/GCE (d), TiO_2_/GCE (e), and bare GCE (f) as illustrated in Figure 4B. As can be seen, the CV of ACV shows a broad peak and poor current response at bare GCE, revealing sluggish electron-transfer kinetics. At TiO_2_/GCE and rGO/GCE, the peak current increases due to the huge surface area of nanomaterials. In great contrast, a well-defined and resolved oxidation peak of ACV can be observed at rGO–TiO_2_/GCE, rGO–Au/GCE, and rGO–TiO_2_–Au/GCE. Most notably, the rGO–TiO_2_–Au/GCE shows the highest augmentation toward the determination of ACV. The outstanding electrochemical response may be due to the large surface area, excellent electrical conductivity, and remarkable electrocatalytic activity of rGO–TiO_2_–Au nanocomposites, which provided the fastest electron transport at the electrode surface. These results clearly indicate that rGO–TiO_2_–Au/GCE is very suitable for the determination of ACV.

### Quantitative Analysis of Acyclovir by Using rGO–TiO_2_–Au/GCE

**FIGURE 5:**
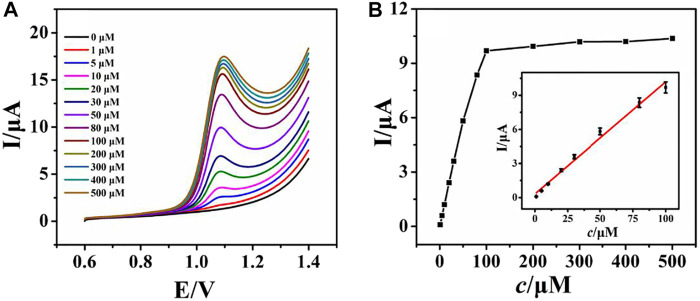
LSV curves of various concentrations of ACV (0–500 µM, down to up) at rGO–TiO_2_–Au/GCE in pH 6.0 PB solution **(A)**; plots of the peak current against the concentration of ACV **(B)**. Inset: the calibration plots for ACV.

Under optimized conditions (see Electronic Supplementary Material), LSV was recorded by varying the concentration of ACV at the rGO–TiO_2_–Au/GCE (Figure 5). Figure 5A shows the LSV responses of rGO–TiO_2_–Au/GCE for varying the concentration of ACV from 0 to 500 μM in 0.1 M PB solution. As illustrated, the successive increase of the peak current is observed by raising the concentration of ACV. The plots of peak current responses against ACV concentrations are shown in Figure 5B. It can be seen that the peak current responses of ACV increased linearly with its concentrations from 1 to 100 μM. For higher concentrations, a deviation from linearity is observed, which is due to the adsorption of ACV or its oxidation product on the electrode surface. The linear regression equation is Ipa (µA) = 0.1403*c* + 2.1814 (µM) with a correlation coefficient of 0.996 (Figure 5B inset). The detection limit is 0.3 μM based on a signal-to-noise ratio of 3. The rGO–TiO_2_–Au/GCE showed a comparable or better dynamic range and detection limit for ACV compared to other electrochemical sensors, such as Cu nanoparticle-modified carbon paste electrode (2.64 μM) (Heli et al., 2010), pencil graphite electrode (0.3 μM) (Dilgin and Karakaya, 2016), nanoporous nickel microsphere-modified carbon paste electrode (40 μM) (Heli et al., 2012), fluorine-doped SnO_2_ electrode (1.25 μM) (Martínez-Rojas et al., 2017), and CdO/Fe_3_O_4_-modified carbon paste electrode (0.3 μM) (Naghian et al., 2020).

### Reproducibility, Repeatability, Stability, and Selectivity of rGO–TiO_2_–Au/GCE

**FIGURE 6:**
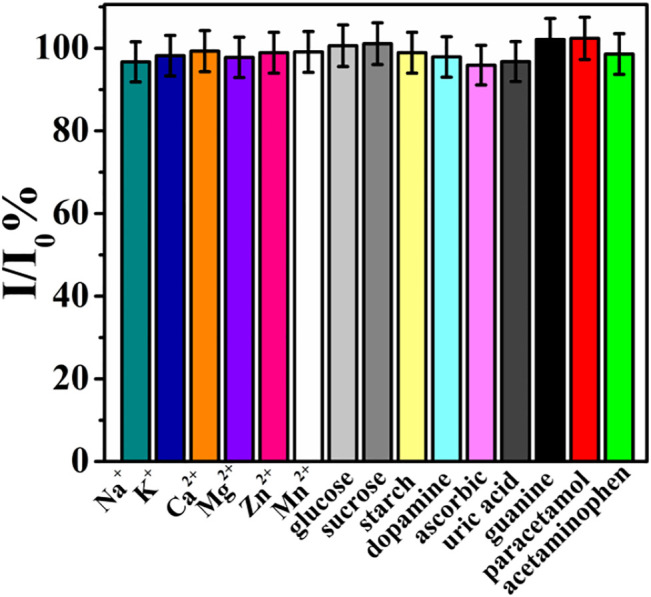
Current ratio (I/I_0_) of rGO–TiO_2_–Au/GCE with 100 μM ACV in the presence of possible interfering species.

To evaluate the reproducibility of rGO–TiO_2_–Au/GCE for the analysis of ACV, five rGO–TiO_2_–Au/GCEs were prepared independently with the same fabrication procedure, and their peak current values toward 100 µM ACV were compared. A relative standard deviation (RSD) of 3.18% was obtained, demonstrating the excellent reproducibility of the proposed sensor. The repeatability of rGO–TiO_2_–Au/GCE was estimated by performing five successive measurements with the same rGO–TiO_2_–Au/GCE in the same solution. It was observed that the oxidation peak current of ACV decreased continuously, which was due to the adsorption of an oxidative product of ACV on the electrode surface. In this case, rGO–TiO_2_–Au/GCE can only be used for a single measurement. Meanwhile, the stability of TiO_2_–Au–rGO/GCE was studied by storing the electrode in a refrigerator at 4°C. After two weeks, the peak current signal of the electrode remained at 90.2% of its initial response, suggesting the good storage stability of the modified electrode. The selectivity of rGO–TiO_2_–Au/GCE toward the analysis of ACV was evaluated by testing some possible interfering species, including Na^+^, K^+^, Ca^2+^, Mg^2+^, Zn^2+^, Mn^2+^, glucose, sucrose, starch, dopamine, ascorbic acid, uric acid, guanine, paracetamol, and acetaminophen. As shown in Figure 6, a 100-fold concentration of each species had almost no influence on the peak current of ACV with deviations below 5%. These data revealed the good selectivity of rGO–TiO_2_–Au/GCE toward the determination of ACV.

### Analysis of a Real Sample

The practical application of rGO–TiO_2_–Au/GCE in the analysis of real samples was studied by determination of ACV in commercial ACV tablet samples. The procedures for the analysis of tablets were followed as specified in *Analysis of the Sample*. The sample was spiked with three levels of ACV in the calibration range, and the content of ACV in the tablet samples was calculated by the standard addition method keeping the dilution factor in consideration. The result shows that the content of ACV was 196.2 mg per tablet, which was very close to the claimed value of 200 mg per tablet. Moreover, the recovery tests of ACV were performed, and the results are presented in Table 1. The satisfactory sample recoveries indicated the validity of the developed sensor for the determination of ACV in pharmaceutical formulations.

**TABLE 1:** Determination of ACV in commercial ACV tablet samples (*n* = 3).

Sample	Added (µM)	Found (µM)	Recovery (%)	RSD (%)
1	20.00	19.19 ± 0.08	96.0	0.88
2	50.00	47.45 ± 0.15	94.9	0.29
3	80.00	85.67 ± 0.24	107.1	1.24

## Conclusion

In this work, the sensitive determination of ACV was achieved by using the rGO–TiO_2_–Au/GCE. The formation of rGO–TiO_2_–Au nanocomposites was verified by TEM, XRD, and XPS techniques. The electrochemical experiments demonstrated that the rGO–TiO_2_–Au/GCE showed outstanding electrocatalytic activity for the oxidation of ACV in pH 6.0 PB solution. Based on the unique properties of rGO, TiO_2_, Au, and their synergistic effects, a good linear detection range and low detection limit for ACV were achieved. The rGO–TiO_2_–Au/GCE also represented acceptable selectivity, repeatability, and stability and offered satisfactory recovery when applied for the analysis of ACV in the real samples.

## Data Availability

The original contributions presented in the study are included in the article/Supplementary Material, further inquiries can be directed to the corresponding authors.
