# Supplementary material for: Improved Performance for the Electrochemical Sensing of Acyclovir by Using the rGO–TiO2–Au Nanocomposite-Modified Electrode
Source: Front Chem. 2022 May 11;10:892919. doi: 10.3389/fchem.2022.892919 (PMC9130495; doi:10.3389/fchem.2022.892919)
Supplement: Supplementary file 1 [file DataSheet1.docx]

**Electronic Supplementary Material**

**Improved performance for electrochemical sensing of acyclovir by using rGO-TiO_2_-Au nanocomposites modified electrode**

Xin-Yang Lu, Jing Li, Fen-Ying Kong*, Mei-Jie Wei, Pei Zhang, Ying Li, Hai-Lin Fang, Wei Wang*

School of Chemistry and Chemical Engineering, Yancheng Institute of Technology, Yancheng 224051, China

**Fig. S1.** The possible reaction mechanism of ACV at rGO-TiO_2_-Au/GCE.

**Optimization of experimental conditions**

To obtain the best electrochemical sensing performance of rGO-TiO_2_-Au/GCE for the determination of ACV, several factors affecting the current response of ACV at rGO-TiO_2_-Au/GCE were optimized.

*Effect of the supporting electrolyte pH*

The effect of solution pH on the electrochemical responses of ACV at rGO-TiO_2_-Au/GCE was investigated in PB solution with pH ranging from 5.5 to 8.0. The plots of different pH versus oxidation peak currents and peak potentials obtained from CVs is shown in Fig. S2. As can be seen, the oxidation peak potential of ACV shifted linearly to more negative potentials with the increase of pH values (Fig. S2A), illustrating that the proton is involved in the electrode process. According to the structure of ACV, the electrochemical oxidation process of ACV is an equal number of protons and electrons. Moreover, the oxidation peak currents of ACV increase from 5.5 to 6.0, and then decline continually with further increasing pH value (Fig. S2B). The maximum value of peak currents was observed at pH 6.0. So, pH of the PB solution was selected as 6.0 for all subsequent electrochemical measurements.

**
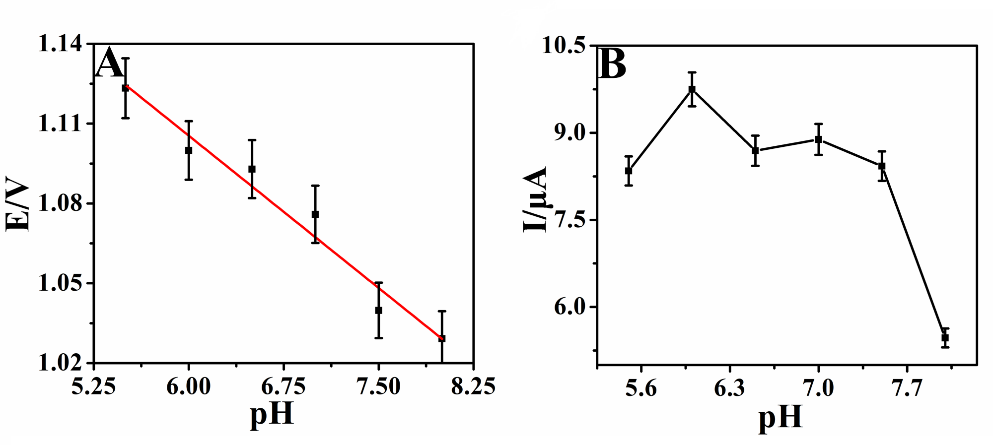
**

**Fig. S2.** Effect of different pH (5.5–8.0) on the oxidation peak potential (A) and peak current (B) of ACV at rGO-TiO_2_-Au/GCE.

*Effect of scan rate*

The effect of scan rate on the oxidation response of ACV at rGO-TiO_2_-Au/GCE was subsequently explored by CVs (Fig. S3). As seen in Fig. S3A, with the increase of scan rates from 5 to 400 mV s^−1^, the oxidation peak current of ACV increases. The relationship between the oxidation peak currents of ACV and the scan rate is obtained to be linear with the scan rate over the studied range (Fig. S3B), suggesting that the oxidation of ACV is controlled by adsorption of ACV to the electrode surface. In addition, oxidation peak potential of ACV shifts positively when gradually increasing scan rate, implying an irreversible oxidation process of ACV.


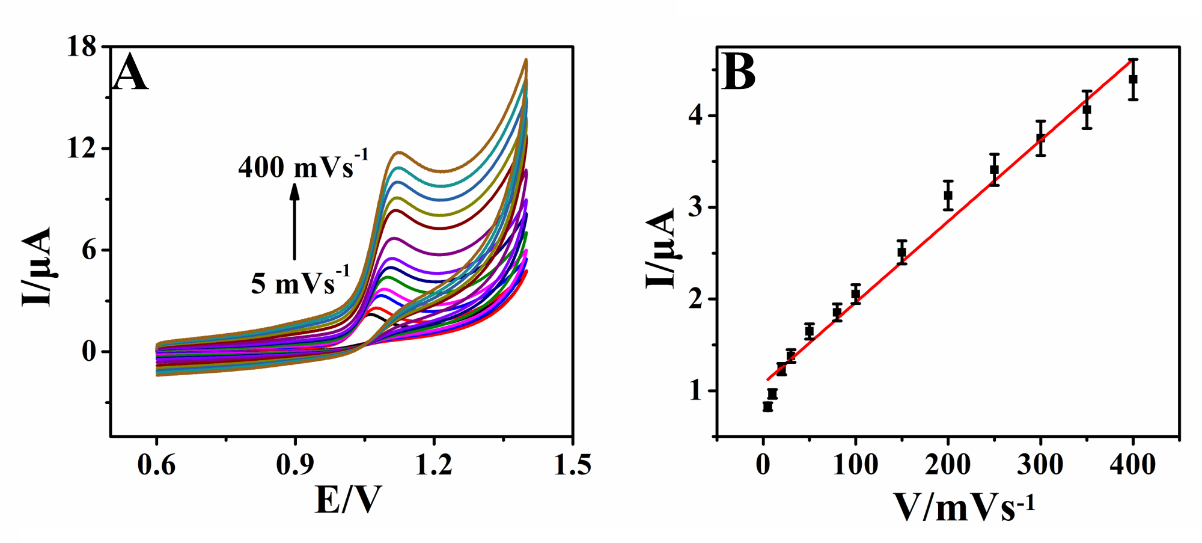


**Fig. S3.** Effect of different scan rate (5–400 mV s^−1^) on the oxidation peak current of ACV at rGO-TiO_2_-Au/GCE in 0.1 M PB (pH 6.0) (A); Plots of oxidation peak current of ACV versus scan rate (B).

*Effect of modifier amount*

The effect of the amount of rGO-TiO_2_-Au suspension on the oxidation peak current of ACV was evaluated. For this purpose, various volumes of the rGO-TiO_2_-Au suspension were deposited onto the electrode surface. It is observed from the Fig. S4 that the increase in the volume of rGO-TiO_2_-Au suspension from 3 to 7 μL resulted in an increment in the oxidation peak current of ACV. When the volume of rGO-TiO_2_-Au suspension beyond 7 μL, the oxidation peak currents of ACV gradually decreased. Hence, the optimum volume of rGO-TiO_2_-Au suspension for the determination of ACV is 7 μL.





**Fig. S4.** Effect of amount of rGO-TiO_2_-Au suspension on the oxidation peak current of ACV in 0.1 M PB (pH 6.0).

*Effect of accumulation time and potential*

For adsorption-controlled reactions, accumulation time and potential are important factors which affected the sensor performance. Bearing this in mind, the effect of accumulation time and potential on the oxidation peak current of ACV were studied at rGO-TiO_2_-Au/GCE. As shown in Fig. S5, increasing the accumulation time from 0 to 80 s, the oxidation peak current of ACV rapidly enhances. When further extending the accumulation time from 80 to 300 s, the oxidation peak current of ACV decreases, which indicates that the amount of ACV adsorption on the electrode surface has been saturated. In addition, when changing the accumulation potential, the oxidation peak current of ACV changes a little. Hence, accumulation step was performed under open-circuit for 80 s.





**Fig. S5.** Effect of different accumulation time (0–300 s) on the oxidation peak current of ACV at rGO-TiO_2_-Au/GCE in 0.1 M PB solution (pH 6.0).
